# Prevalence of cauliflower ear in high level judoka

**DOI:** 10.1038/s41598-023-42635-8

**Published:** 2023-10-13

**Authors:** Andreas Nitsch, Hannes Marx, Cornelius S. Fischer, Sinan Bakir, Axel Ekkernkamp, Georgi I. Wassilew, Lyubomir Haralambiev

**Affiliations:** 1 Center for Orthopaedics, Trauma Surgery and Rehabilitation Medicine, University Medicine Greifswald, Greifswald, Germany; 2 BG Trauma Center Tübingen, Department of Traumatology and Reconstructive Surgery, Eberhard Karls University Tübingen, Schnarrenbergstraße 95, 72076 Tübingen, Germany; 3 Department of Trauma and Orthopaedic Surgery, BG Klinikum Unfallkrankenhaus Berlin, Berlin, Germany

**Keywords:** Trauma, Risk factors

## Abstract

Judo is an Olympic sport, and the way of its performing can lead to repetitive blunt injuries on head and ears. The chronic consequences of such traumata on the auricle are the formation of so-called cauliflower ear. This condition is painful, can lead to interruptions in the training process and long-term consequences for the athlete's health. There is limited knowledge of epidemiological data about cauliflower ear deformities in judo. Evaluation of the prevalence of cauliflower ear among judokas based on their profile pictures on the international judo federation was performed. A large cohort of judo athletes from around the world was studied. Two different classifications for the severity of ear deformities were used. Statistical calculations of the collected data and correlations to different parameters were performed. Images of 1632 top athletes were evaluated in the study. Ear deformities were found in 55.5% of the judokas. There was gender-specific differences. Male athletes were affected much more often than female athletes. In addition, ear deformities were more pronounced in male athletes. A correlation was found between the age of the athletes and the presence of an ear deformity. It has also been shown that judokas with a high world ranking are more likely to have an ear deformity. Ear deformities are a common consequence of injury among leading judo athletes. The current study represents the largest and high heterogeny cohort ever conducted on the prevalence of cauliflower ear in judoka. Knowledge of the prevalence of cauliflower ear in judoka based on reliable data from this study, may be important prerequisites for further studies on the impact of this traumatic consequence on training preparation and judoka health.

## Introduction

Judo is a worldwide popular martial art and the first of this family of combat sports that became an Olympic discipline^1^. Today judo is organized by the International Judo Federation (IJF) which counts 204 member countries^2^ with more than 40 million people practicing judo around the world^3^. The high popularity of judo also harbors risks for combat-specific injuries. These are defined by the way the sport is carried out. As a martial art, there are no direct punches or kicks in judo, but the opponent must first be grabbed by the judogi (the judo uniform) and then thrown or brought under control on the ground^4^. Prescribed techniques such as throws, holds, chokes, and armlock techniques are to be used to achieve victory Further, a victory can be achieved by penalties of the opponent for forbidden actions, inactivity or avoiding-fight behavior^5^. A number of factors such as physical condition^6–8^, weight loss related to the specific weight division^9^ and technical preparation of the judoka, but also various psychological factors play a role in the issue of competitive performance^8,10^. As a combat sport in judo, whether recreational or competitive, there are a number of injuries that can occur. Some of the injuries may be related to the style of combat. The grip-taking or ground fighting in judo, especially the hold-down and the choke techniques, seem to be predisposing for abrasions, hematoma and injuries in the athlete’s head area^11^. Comparable with wrestling, such numerous shearing forces, pressure, or abrasion forces the head area can affect^12^ also the pinnae, as prominent and unprotected anatomical structures. These injuries often lead to acute hematomas of the auricle^12–15^. The auricular hematoma occurs between the perichondrium and the auricular cartilage, disrupting its blood supply^13,16–18^. Left untreated, this condition caused fibrotic changes^19,20^ and post-traumatic pinna thickening^21–23^. The transformation phase of the subperichondrial hematoma due to the migration of chondroblasts into the new mature cartilage tissue lasts about a month^22^. The clinical picture of this process is the so-called cauliflower ear^20,22,24^, which is also known as a wrestler's ear^19,25^. Some authors are regarding this pinna deformity also as a typical for judo sport^26^, Several studies reports specific the risk of head and face injuries in judo^27–30^. Some of those studies mentioned also injuries of the ears^31^, but there are still very poor data about the prevalence of cauliflower ears in judo^32^. Since the condition of cauliflower ear can be associated with chronical pains, discomfort, and even with hearing impairment of the athletes^32–34^, the question of their prevalence in judo also deserves the attention of sports medicine.

The aim of this study was to evaluate the presence of cauliflower ear deformities in a larger cohort of top-level judo athletes spanning national restrictions. In addition, the question should be answered whether there are gender or weight class differences in the judokas with this ear deformity phenomenon.

## Material and methods

### Athletes and data collection

The prevalence of cauliflower ears was studied using photographs of high level ranked judokas. Only publicly available data from judo athletes from the official IJF website (www.ijf.com) was used to collect the data. This study was carried out after the approval of the ethics committee of the University Medicine Greifswald (BB 064/20). The test results and data on age, gender, weight class, world rankings, and nationality were processed and evaluated anonymously so that they cannot be assigned to individual athletes. For the study, all athletes 18 years of age or older in category of seniors were considered who had a profile with a photo on the IJF website between January 1st, 2021, and February 28th, 2021. To avoid duplication in the evaluation or contradictions caused by updating of data, e.g. weight division or world ranking, the athletes were analyzed by national federations unblock. The daily updated profiles of the judokas in the respective national federation were viewed in alphabetical order by three medical doctors independently of one another. The main aim was to examine the existing image material of the athletes for the presence of a cauliflower deformity and to classify them using two classifications. The results of the independent investigators were then compared and discussed, after classifying the ear’s deformity of an individual athlete. The consensus about the degree of cauliflower ear has been recorded. This procedure was applied further for the next athlete.

Only athletes whose IJF profile contained more than one image, allowing the assessment of both ears, were included in the study. In addition, only profile pictures of the athletes with high resolution have been considered, that also after zooming in its still allowed the adequate evaluation of the pinna. Further exclusion criteria were factors preventing the assessment of the external ear—bad quality of the picture, like poor exposure or sharpness, or also when the ears in the profile picture were covered by the athlete's hair or headscarf or the ears were not fully depicted due to the angle of the photograph or the position of the head.

### Classifications

Each ear of the athletes was evaluated separately, those athletes were also considered in which only one of the auricles could be assessed due to the image morphology.

**Figure 1:**
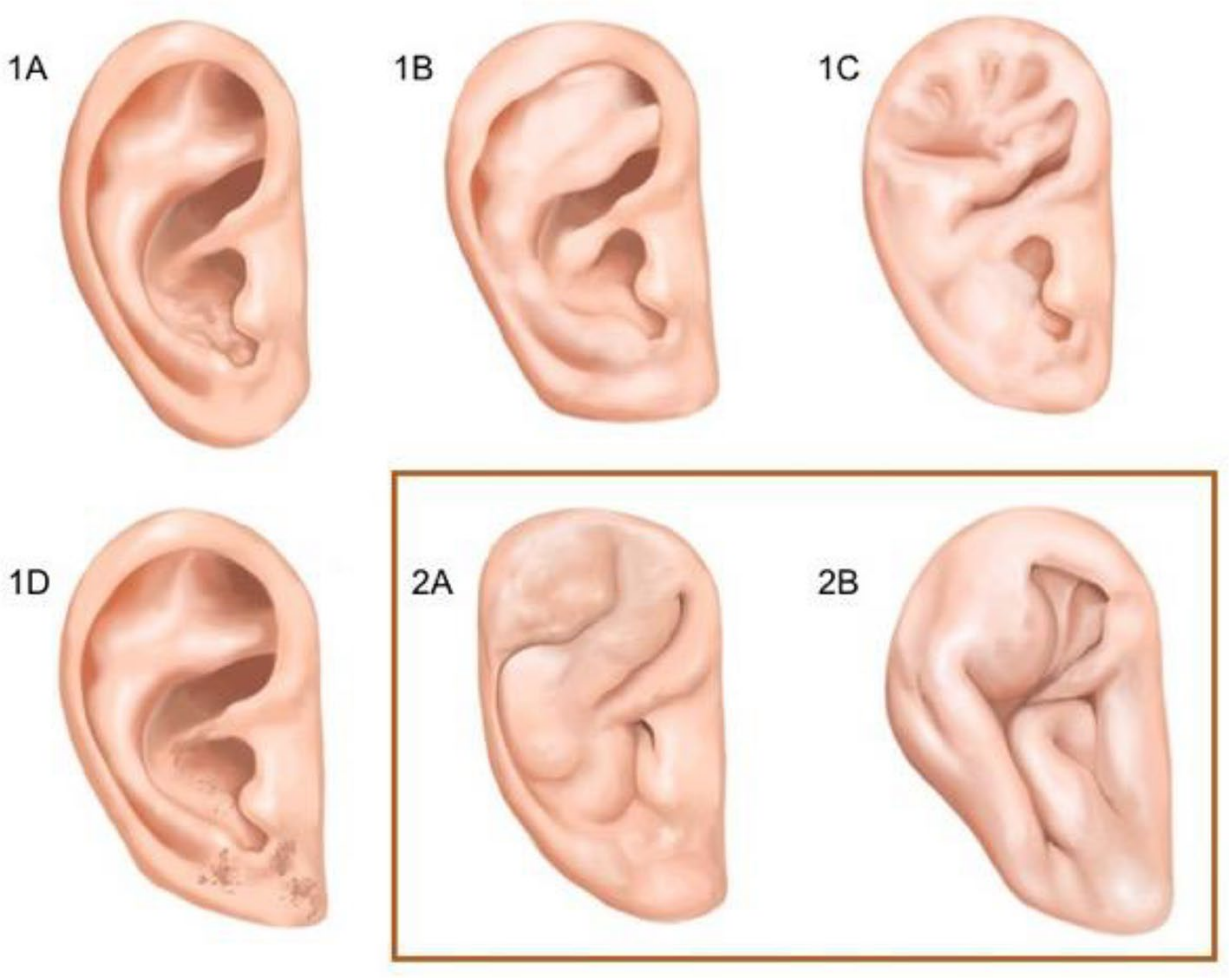
Classification of the deformities of pinna by Yotsuyanagi et al.^35^: Type 1 deformities with no change or only a slight change in the outline of the external ear. Five sub-type were defined: (**1A**)—Deformity restricted to the concha; (**1B**)—Deformity extending from the antihelix to the helix; (**1C**)—Deformity extending throughout the ear; (**1D**)—Deformity accompanied by skin defects. Type 2 (marked in orange) describes the deformities that also affect the auricle outline as a significant change: (**2A**) the ear has good structural integrity; (**2B**) the ear has poor structural integrity.

The following two classifications of cauliflower ear were used in the analysis: The first of the classifications, described by Yotsuyanagi et al.^35^, considered stage 1 deformity without changes in the outline of the ear with five sub-levels, and stage 2 considers deformity with substantial changes in the outline of the ear, it has only two subgroups (Fig. 1).

**Figure 2:**
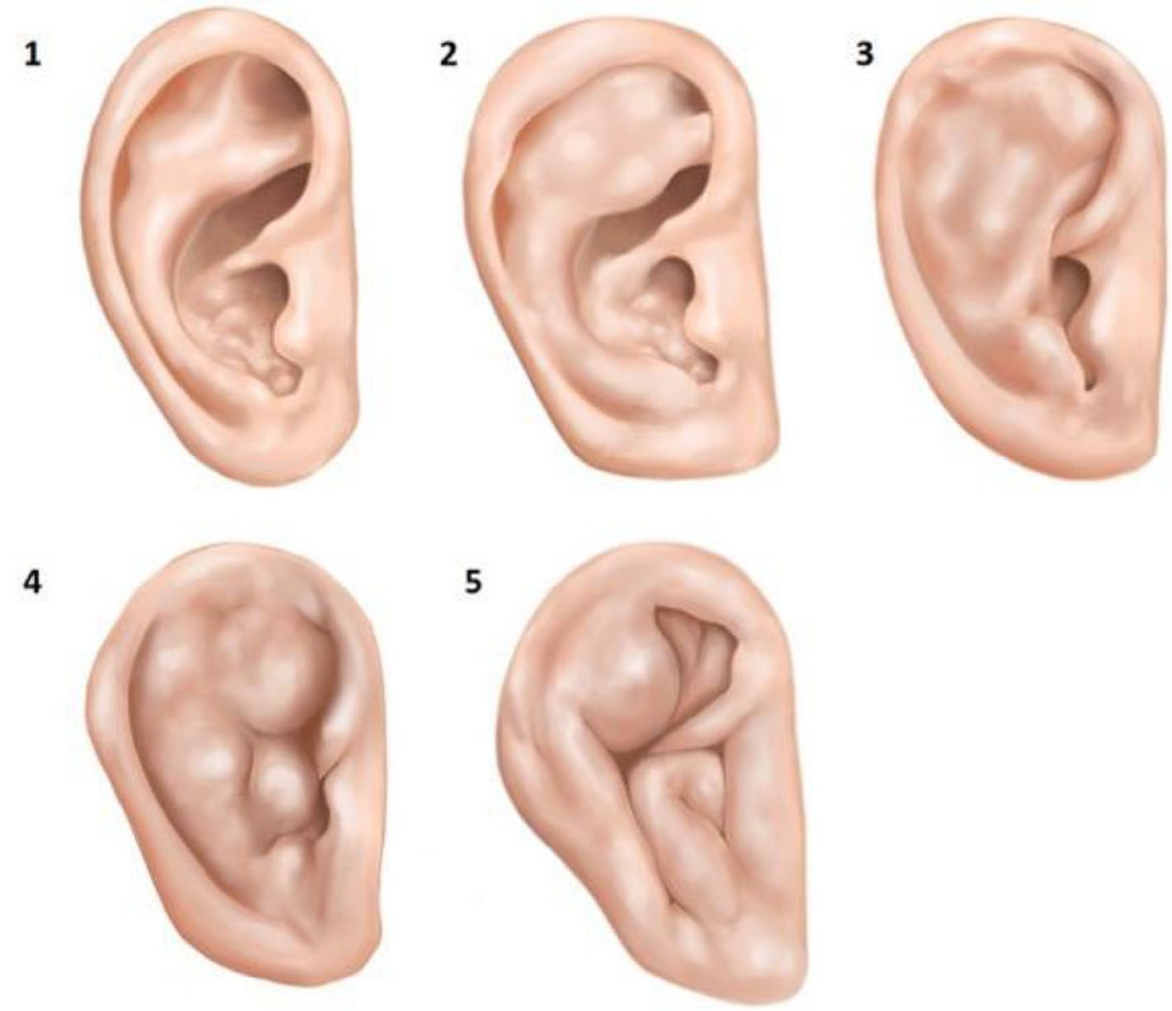
Numerical classification of the severity of pinna deformities^32^: (**1**) minimal deformities of the pinna; (**2**) isolated areas of the auricular morphology are affected—for example only the crura as a part of the antihelix; (**3**) Separate anatomical structures of the auricle are completely affected—the entire antihelix or the entire helix; (**4**) The deformity extends beyond the defined anatomical structures of the pinna and affects several of them; (**5**) "extreme deformity" of the external ear.

In the second classification, the ears were ranked on a scale of increasing severity from 0 to 5, a method used by Manninen et al.^32^. The severity of the auricular deformity is graded in ascending order from 1—minimal changes to 5 "extreme deformity" of the cauliflower ear (Fig. 2).

### Statistics

The data were recorded in tables. The evaluation was carried out with SPSS Version 28. The descriptive statistics were calculated using frequency tables. The graphical processing was done with GraphPad Prism Version 9 or Microsoft Excel 2021. The data were checked for normality using de Shapiro–Wilk's W test. The cross tables were analyzed with Pearson-Chi-Square-Test. Cramer-V was calculated as an indicator of the effect size. Rank correlations were calculated according to Spearman's Rho.

### Ethical approval information

This study was approved by the ethics committee of the University Medicine Greifswald (BB 064/20).

## Results

**Figure 3:**
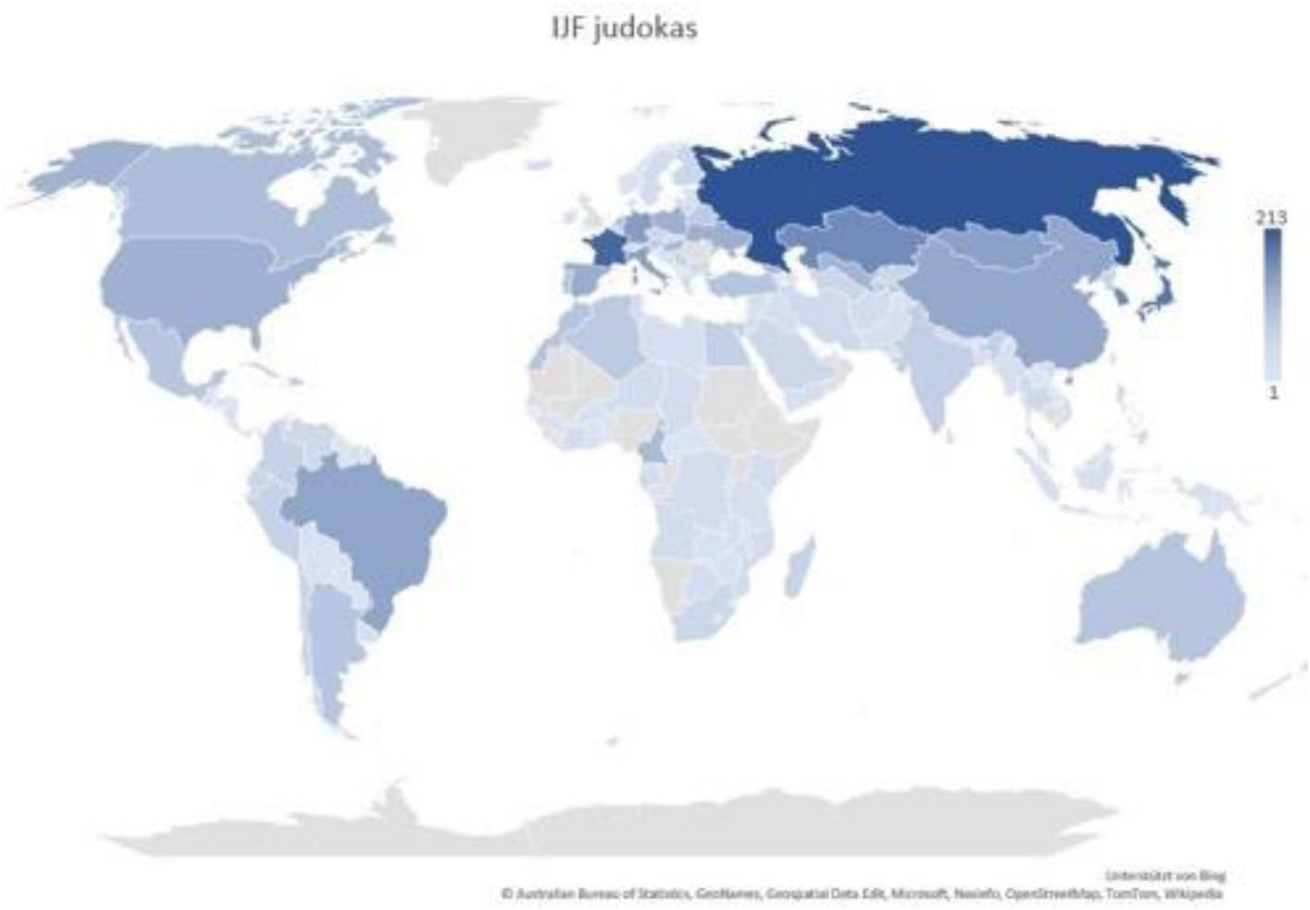
The number of judokas presented on the IJF website by country. The darker the blue color, the higher the number of judokas listed. According to this, the countries in the following descending order have the greatest number of judokas (shown in brackets): the Russian Federation (213), France (184), Japan (162), the Republic of Korea (134), Kazakhstan (129) and Germany (105). The countries that have no entries on the IJF website are gray. This figure was created using the “area cartogram” chart type of Microsoft Excel (version 2021).

There were 3908 judokas from 169 countries presented with pictures on the IJF website—2448 male (62.6%) and 1459 females (37.3%). The national judo federations are organized into five continental unions: the African Judo Union (AJU) with 54 countries and 399 judokas (10.2%), the Judo Union of Asia (JUA) with 43 countries, and 1097 judokas (28.1%), the European Judo Union (EJU) with 54 countries and 1780 judokas (45.5%), the Oceania Judo Union (OJU) with 20 countries and 65 judokas (1.7%), and the Panamerican Judo Confederation (PJC) with 36 countries and 556 judokas (14.2%). The countries with the highest number of judokas listed (Fig. 3) were the Russian Federation with 213 (5.5%), France with 184 (4.7%); Japan with 162 (4.1%); the Republic of Korea with 134 (3.4%); Kazakhstan with 129 (3.3%) and Germany with 105 (2.7%) athletes.

Not all of these judokas could be included in the study. About one-fifth of them had only one profile picture published, which was insufficient for assessing an ear deformity. About one-third of the athletes had to be excluded because the ears were obscured or not fully depicted on their profile pictures. In about 10% of the cases, the images were not evaluable due to poor quality (resolution, exposure, sharpness, etc.).

After applying the exclusion criteria on the available images, a total of 1632 judokas were included in the study and their IJF profile photos has been assessed, 1014 male (62.1%) and 618 females (37.9%). They were between 18 and 41 years old (mean age 25.6 years SD ± 4.00), for 9 athletes there was no age given on the homepage.

The distribution across the weight divisions of the judokas was almost homogeneous for both men and women. The heaviest weight divisions had the lowest number of judokas (Table 1).

**Table 1:** Athletes included and their weight divisions.

Male			Female		
Weight division	N	%	Weight division	N	%
− 60	146	14.4%	− 48	95	15.4%
− 66	161	15.9%	− 52	101	16.3%
− 73	167	16.5%	− 57	97	15.7%
− 81	177	17.5%	− 63	90	14.6%
− 90	148	14.6%	− 70	90	14.6%
− 100	106	10.5%	− 78	76	12.3%
+ 100	106	10.5%	+ 78	68	11.0%
Missing	3	0.3%	Missing	1	0.2%
Total	1014	100.0%	Total	618	100.0%

**Figure 4:**
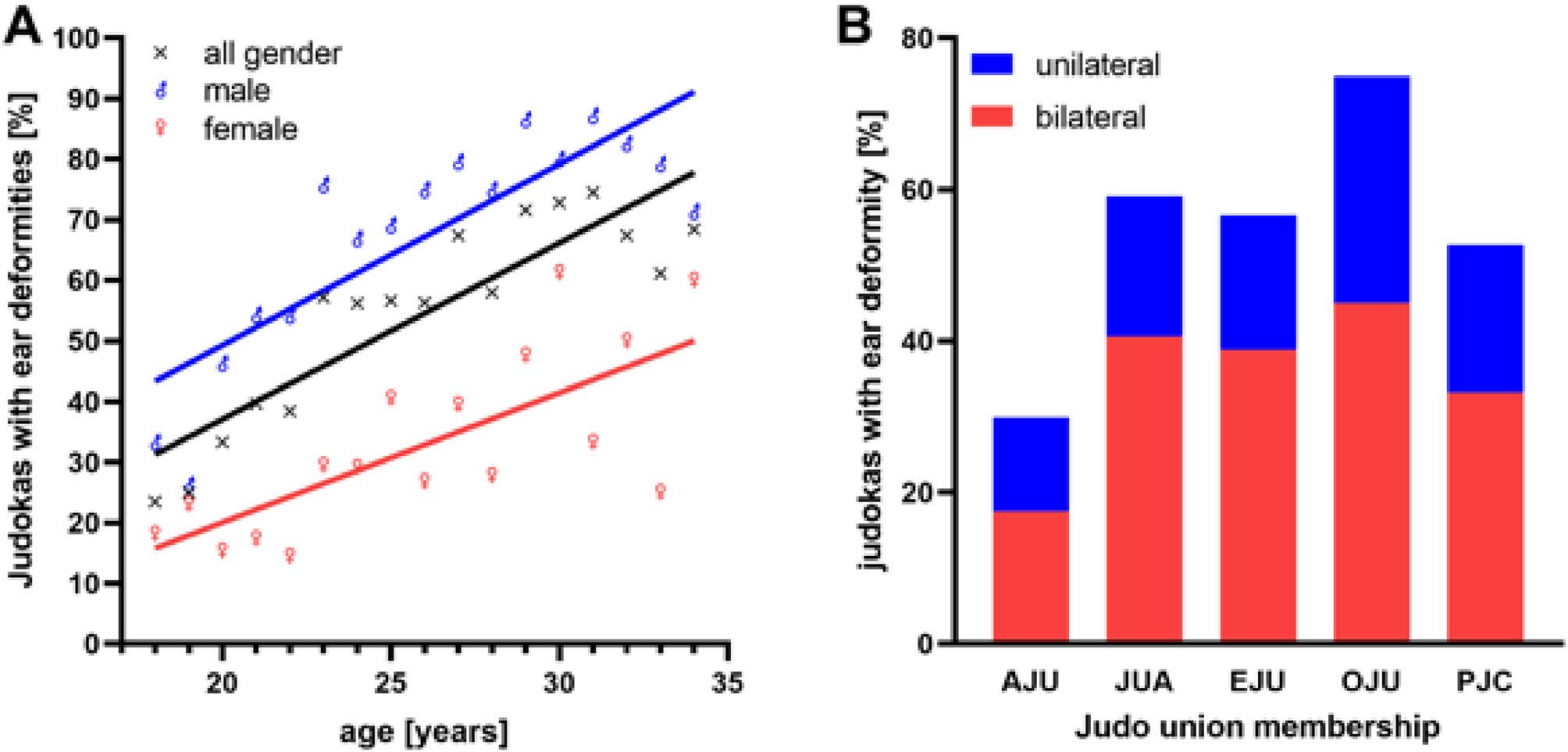
Correlation between the age of the judokas and the prevalence of cauliflower ears and frequency of ear deformities in different judo unions. (**A**) Increasing age of athletes correlated with a higher prevalence of cauliflower ear (black line). This was true for both male (blue line) and female athletes (red line). (**B**) Presence of a unilateral (blue) or bilateral (red) ear deformity in the different judo unions.

An ear deformity was found in 55.5% of the judokas, of which 32.4% were unilateral and 67.6% bilateral. There was a significant correlation between the age of the judokas and the occurrence of cauliflower ears (ρ = 0.889, p < 0.001). The older the athletes were, the more common the auricular deformity was. The relationship between increasing age and an increasing probability of ear deformity was found in both male (ρ = 0.797, p < 0.001) and female ρ = 0.721, p < 0.001) athletes (Fig. 4A).

There was a significant connection between Union membership and the prevalence of cauliflower ears in the judokas (χ^2^ = 34.830, p < 0.001, V = 0.103).

The AJU judokas had the fewest cauliflower ear deformities—12.4% of those had unilateral and 17.5% bilateral. Athletes from the OJU were most often affected—here 30.0% had a unilateral and 33.2% a bilateral ear deformity. The frequency of ear deformities was 56.4% among members of the EJU, of which 17.8% were unilateral and 38.8% bilateral. Among the athletes from the JUA, 59.2% had ear deformities, 18.5% unilaterally, and 40.6% bilaterally. 52.7% of the judokas of PJC had ear deformities—19.5% unilateral and 33.2% bilateral (Fig. 4B).

### Severity cauliflower ear

The right and left ears were analyzed separately using both scores.

Image analysis of the right ear showed the following: according to Yotsuyanagi's classification^35^, 1C (51.5%) and 1B (44.1%) were assigned most frequently. According to the numerical scale (0–5), grades 3 (29.8%) and 2 (26.0%) occurred most frequently.

The results were similar for the left ear: According to Yotsuyanagi's classification^35^, 1C (54.1%) and 1B (43.4%) occurred most frequently. Classified according to the numeral scale, grades 3 (30.2%) and 2 (26.1%) were the most common. The right and left ear were affected to about the same extent by deformities.

### Gender and cauliflower ear prevalence

The correlation between gender and frequency of cauliflower ear was examined. There was a significant difference in the prevalence of auricular defects in males and females (χ^2^ = 231.242, p < 0.001, V = 0.376). While 68.4% of female judoka showed no deformities of the auricle, this was the case in only 29.9% of male athletes. Unilateral cauliflower ear deformities were found in 17.3% of women and 18.4% of men. Bilaterally, 14.2% of women and 51.7% of men were affected.

**Figure 5:**
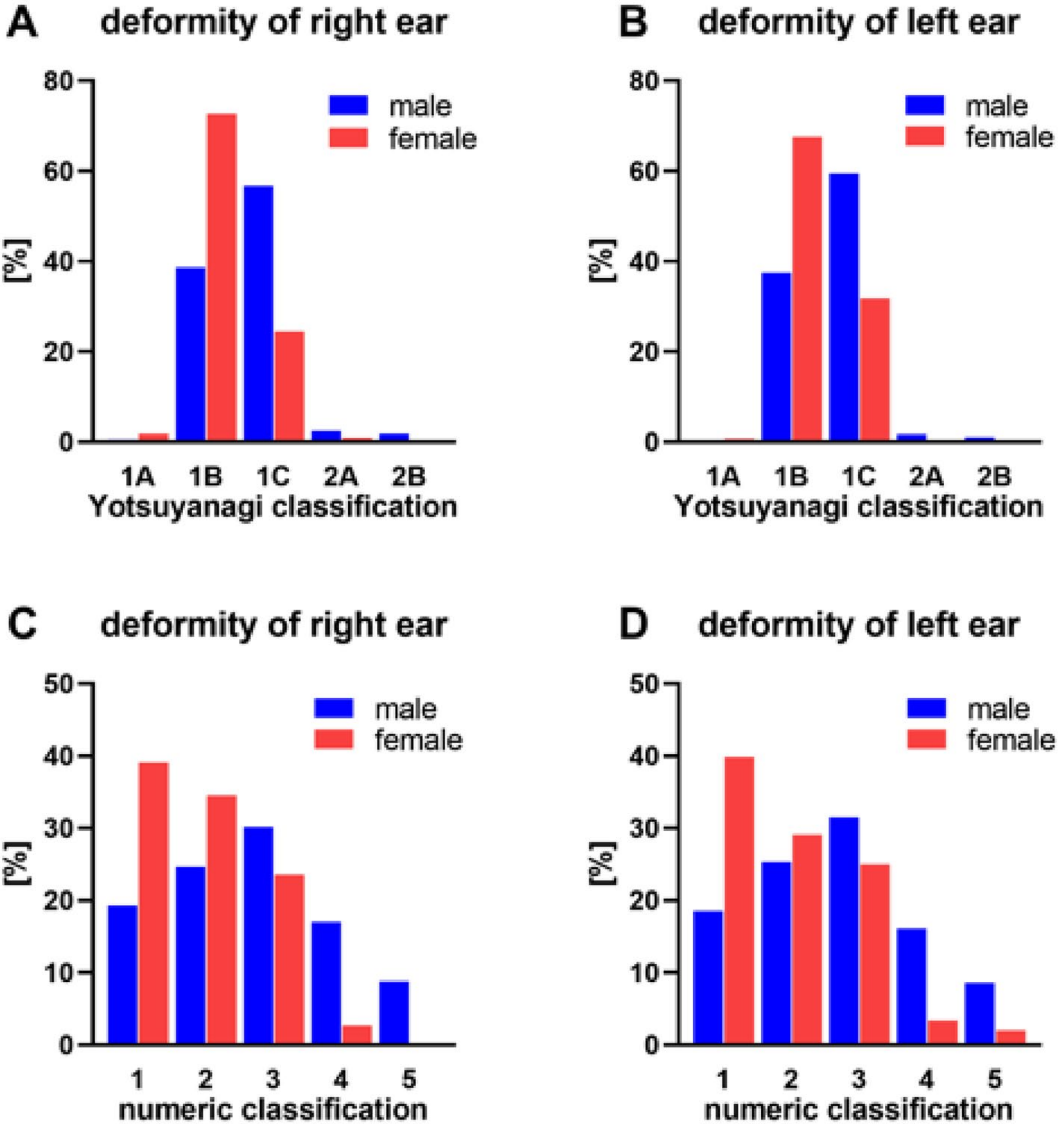
Gender specific difference between the severity of cauliflower ears. Classification and severity of the observed ear deformities according to Yotsuyanagi et al.^35^ (**A** + **B**) and according to a numerical scale (**C** + **D**). Analyzed separately for the left and right ears in male (blue) and female (red) athletes. (**A**) Increasing age of athletes correlated with a higher prevalence of cauliflower ear (black line).

In view of the severity of the deformity, there was a gender-specific difference. Regardless of side or classification, ear deformities were more severe in male athletes. According to Yotsuyanagi’s classification (Fig. 5A + B), male athletes had a higher severity of cauliflower ear deformity than females (right ear: χ^2^ = 266.405, p < 0.001 V = 0.409; left ear: χ^2^ = 240.382 p < 0.001, V = 0.389). In addition, according to the numerical classification of the cauliflower ear (Fig. 5C + D), males showed higher degrees of deformity than females (right ear: χ^2^ = 254.772, p < 0.001 V = 0.400; left ear: χ^2^ = 243.026 p < 0.001, V = 0.392).

In the male judokas, the deformities were most frequently assigned to category 1C. On the other hand, the deformities of the ears of the female athletes were most frequently assigned to category 1B, one level lower. According to the Manninen classification, this difference was even greater. Here, category 3 deformities were most common among athletes. Deformities in the lowest category 1 were most common among female athletes, two levels lower.

With regard to the reasons for exclusion, there was also a gender-specific difference (right ear χ^2^ = 841,012, p < 0.001, V = 0.464, left ear: χ^2^ = 867.327, p < 0.001, V = 0.471). In about one-third of the women, the ears were covered by long hair and could not be used for the analysis (left ear: 35.6%; right ear: 35.2%). For male athletes, this proportion was less than 4%. A few female athletes, who otherwise had images suitable for evaluation, wore a headscarf on the pictures and could not be used for evaluation. None of the male athletes wore a headscarf.

### Weight division and cauliflower ear

**Figure 6:**
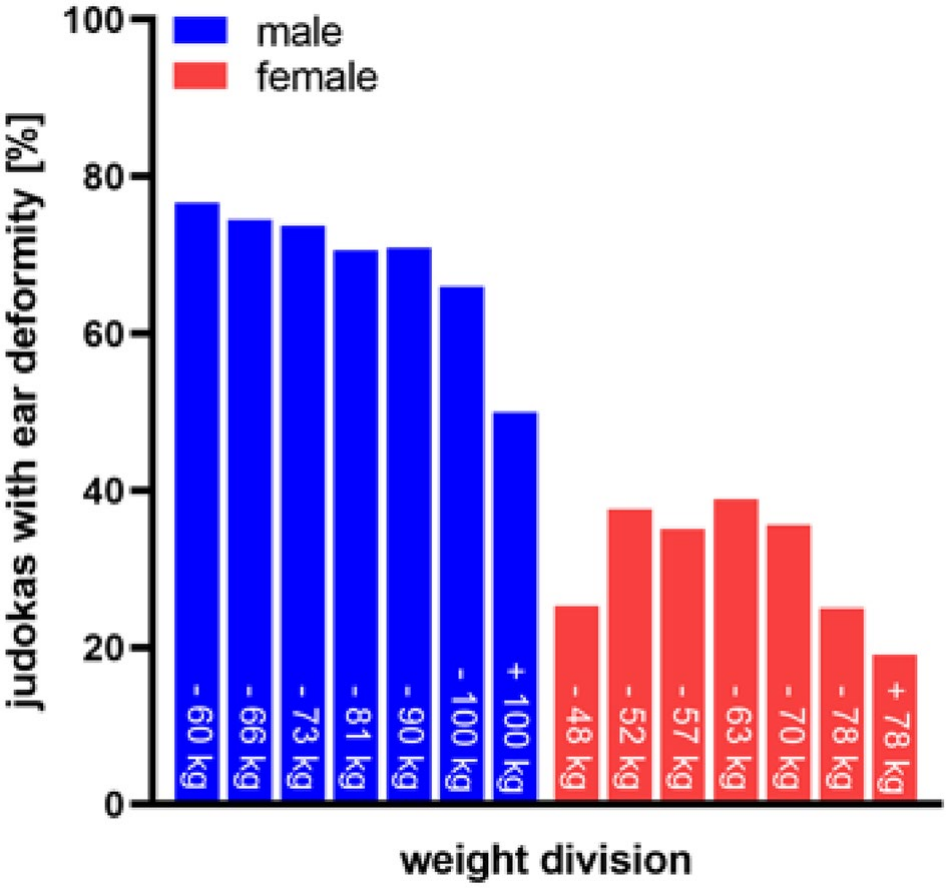
Prevalence of cauliflower ears in different weight divisions. Frequency of ear deformities depending on the weight class of male (blue) and female (red) athletes.

The presence of cauliflower ears in judoka in different weight divisions was investigated (Fig. 6). There was a significant correlation between ear deformity and weight division (male: χ^2^ = 29.773, p < 0.01, V = 0.172, female: χ^2^ = 26.053, p < 0.05, V = 0.145). The most common ear deformities in male athletes showed the lightest weight divisions 76.7% (20.5% unilateral and 56.2% bilateral). There was a tendency that cauliflower ears were less common among male athletes in higher-weight divisions. In the weight division over 100 kg only 50.0% of athletes were affected by ear deformities (16.0% unilateral, 34.0% bilateral). Among female athletes, ear deformities were most common in the middleweight division (up to 63 kg), at 38.9%. In the higher and lower weight divisions, the prevalence of deformity was lower.

The relationship between the degree of deformity and weight division was examined. The analysis of the numerical classification of the cauliflower ear showed that the higher the weight class, there is a slight tendency to decrease the degree of deformity (right ear: ρ = − 0.089, p < 0.01, left ear: ρ = − 0.095, p < 0.01).

### World rank list and cauliflower ear

A high world ranking by the judokas correlates with a higher cauliflower ear presence (ρ = − 0.136, p < 0.01).

Similar correlations are also found in the world ranking of athletes and the severity of pinna deformities (right ear: ρ = − 0.124, p < 0.01, left ear: ρ = − 0.116, p < 0.01). The higher the judoka's position in the world rankings, the greater the degree of deformity.

## Discussion

Information about the prevalence of auricular deformities in judo athletes exists only in isolated reports^32^ and therefore, there is a lack of meaningful data on these sports injuries. The specifics of judo performance seem to be relevant to head injuries and, respectively, to favoring the development of ear injuries. The mechanisms of auricle injury are reported primarily as shearing forces and pressure in the athlete's head area^5^. Such situations in judo occur primarily through ground fighting. Analyzes of judo competitions suggest that groundwork can be crucial to an athlete's success. Certain ground holds such as Yoko-Shihō-Gatame showed the highest efficiency index among female fighters^36,37^. For reasons of study design, no correlations can be made between specific judo performance situations and ear injuries in the current study, but these raise interesting questions for further studies. The data collection approach in this study, using public images of athletes on the IJF website, allowed broad access to a large number of judoka without restriction to a specific national cohort. Furthermore, this approach made it possible to carry out the study even in times of the global corona pandemic, in which neither large sporting events nor broad clinical examinations of athletes were possible.

The presentations of each national judo federation on the IJF website varied greatly. Only a few nations hat no athletes’ profiles. Although from all 3 908 Athletes’ profiles, due to missing or bad quality of pictures, only 47.3% (1632) could be considered, this study represents the largest studied population of judokas to date. About two-third of the judokas were male. The five continents have presented wide variations in the number of judokas, which may also be an indication of the different popularity of this sport in these regions of the world. However, it must be taken into account that the continental judo unions differ greatly in the number of member countries and their populations, and these factors also affect the number of judokas. Thus, almost half of the judokas came from the 54 countries of the EJU as the leader, while the number of judokas from the 20 countries of the OJU was very low with 65 athletes. Athletes from the OJU were most frequently affected—30.6% had a unilateral and 36.1% a bilateral ear deformity. It should also be mentioned that the Oceanian Judo Union with a total of 36 athletes did not form a representative subgroup.

This study showed a prevalence of cauliflower ears in judokas of 55.5%, with 32.4% unilateral and 67.6% bilateral deformities. Only one study was found that described the prevalence of cauliflower ear in a small population of 31 Finnish judokas^32^. Comparable results of cauliflower ear prevalence (58%) were reported (33% unilateral and 66% bilateral).

A significant correlation was found between the age of judokas and the occurrence of cauliflower ear deformities, such that as the age of judokas increased, cauliflower ear deformities occurred more frequently. This correlation was found for both male and female judokas. This can be explained by the longer exposure and more frequent injuries to the auricle during longer sports careers.

Considering the gender of the judokas and the frequency of cauliflower ears, there were significant differences in favor of the female athletes. While 68.4% of the women did not show any deformities of the auricle, only 29.9% of the men did. Not only the frequency but also the severity of cauliflower ears is significantly higher in males than in females. Considering the general risk of injuries in judo no relevant differences have been shown between sexes^26^ therefore this cannot explain the differences in the prevalence of cauliflower ear. One possible explanation would be that cauliflower ear is generally more acceptable to male athletes since it is considered a natural consequence of their sport^32^.

Another factor in a higher prevalence of cauliflower ear was the higher position of judokas in the world rankings. The reason for this can also be assumed to be the intensive sporting experience that these athletes have in order to get a higher world ranking. The weight division, on the other hand, had no impact on the prevalence of ear deformities.

The presence of cauliflower ears in athletes is also dependent on proper medical management of recent pinna injuries such as hematomas^21,22,24^. The current study cannot provide any information about the treatments of auricular deformities that have taken place in judokas. Studies in other judo-like sports such as wrestling showed that the willingness to treat their pinna injuries depends on the athletes themselves and varies in different populations. For example, Iranian wrestlers received 23% of some type of treatment for their cauliflower ear^33^, while a group of Finnish athletes received medical treatment in 96%^32^.

However, the authors' conclusion from the Finnish study that the therapy does little to prevent the formation of cauliflower ears is controversial.

In general, there is a lack of knowledge about the treatment of cauliflower ears in the context of sports medicine. Further studies on athletes' acceptance of cauliflower ears and their willingness to seek treatment are needed. Furthermore, studies on the influence of cauliflower ears not only on the athletes' training and competition processes are valuable, but also on their aesthetic and health consequences.

### Limitation

The analysis of the ear deformities performed on pictures, and not performing clinical examinations on the athlete’s ears was connected to some limitations. In case of poor picture’s quality, like low resolution or poor view of the pinna led to difficulties or failure in the classification of the ear deformities. Athletes with such pictures have been excluded from the study. Furthermore, the prevalence of cauliflower ears, but no information about their mechanism of occurrence, or their impact on the athlete’s health and trainings process could be regarded. Prehistory of injuries on pinna were not available and not regarded to the analysis of this ear deformities.

### Guidelines for future studies

The present proposal for further studies is formulated to answer the important questions about the incidence and conditions of occurrence of ear injuries in judo, as well as the actual clinical impact on the athletes.

1. Studies should include not only inspection but also clinical examinations of the athlete's ears.
  This can also enable the detection of smaller hematomas or deformities and improve the validity of the data.
2. Using the numeric classification for ear deformities.
  This enables better comparability of the results if they are then different.
3. Collecting data on localization of ear injuries, frequency of ear hematoma recurrence.
4. Interviews with affected athletes about:
  The judo-specific situations in which these ear injuries occurred.
  Complaints from athletes in the acute phase of the injury.
  Dealing with the ear injury and the athletes' desire for therapy.
  Importance of the ear injury/deformity for the training and competition process.
  Residual consequences of this ear deformity.
  Psychological aspects of ear deformity—aesthetic and self-perception consequences.
5. Interviews with medical experts who treat such ear injuries about therapy options, convalescence time, recurrence rate, prophylaxis options.

## Conclusion

The current study is the first to evaluate the prevalence of cauliflower ear among judokas without restriction to any specific national cohort analyzing pictures of 1632 world-level judo athletes from many nations on all continents. Correlations between age, gender, world ranking, weight division of athletes and the prevalence of cauliflower ear in judokas and it’s severity were examined and reported for the first time. Although there was a clear gender-specific difference in favor of the female athletes, the fact that more than half of the judokas have a cauliflower ear, this can also be described as "typical" for the judo sport.

However, questions about the treatment of cauliflower ears and their impact on the health of judo athletes need to be answered in further studies.

## Data Availability

All data to this study are available upon request by the corresponding author.
